# Supplementary material for: Mapping the evolution of fertility support policies in China: A content and instrumental analysis
Source: PLoS One. 2025 Oct 9;20(10):e0332137. doi: 10.1371/journal.pone.0332137 (PMC12510515; doi:10.1371/journal.pone.0332137)
Supplement: S1 Appendix — (ZIP) [file pone.0332137.s001.zip › S1 Appendix. 226 original policy documents/41-国家卫生健康委办公厅关于印发托育从业人员职业行为准则(试行)的通知(FBM-CLI.4.5144767).doc]

**国家卫生健康委办公厅关于印发托育从业人员职业行为准则(试行)的通知**

制定机关： [国家卫生健康委员会](https://www.pkulaw.com/law/chl?Aggs.IssueDepartment=60321&way=textBasic) [机构沿革](javascript:void(0))

发文字号：国卫办人口函〔2022〕414号

公布日期：2022.11.23

施行日期：2022.11.23

时效性： [现行有效](https://www.pkulaw.com/law/chl?Aggs.TimelinessDic=01&way=textBasic)

效力位阶： [部门规范性文件](https://www.pkulaw.com/law/chl?Aggs.EffectivenessDic=XE0302&way=textBasic)

法规类别： [卫生机构与人员](https://www.pkulaw.com/law/chl?Aggs.Category=09602&way=textBasic)

国家卫生健康委办公厅关于印发托育从业人员职业行为准则（试行）的通知
（国卫办人口函〔2022〕414号）

各省、自治区、直辖市及新疆生产建设兵团卫生健康委：
　　为深入贯彻党的二十大精神，认真落实《中共中央国务院关于[优化生育政策促进人口长期均衡发展的决定](https://www.pkulaw.com/chl/1247bc81dccaeed3bdfb.html?way=textSlc)》和《[国务院办公厅关于促进3岁以下婴幼儿照护服务发展的指导意见](https://www.pkulaw.com/chl/434b24b8b5769383bdfb.html?way=textSlc)》（国办发〔2019〕15号），建设一支品德高尚、富有爱心、敬业奉献、素质优良的托育服务队伍，我委研究制定了《托育从业人员职业行为准则（试行）》。现印发给你们，请结合实际，认真贯彻执行。

国家卫生健康委办公厅
　　2022年11月23日

托育从业人员职业行为准则（试行）

　　托育服务事关婴幼儿健康成长，事关千家万户。为进一步增强托育从业人员的责任感、使命感和荣誉感，规范职业行为，特制定本准则。
　　**一、**坚定政治方向。坚持以习近平新时代中国特色社会主义思想为指导，贯彻落实党中央关于托育工作的决策部署。不得有损害党中央权威和违背党的路线方针政策的言行。

　　**二、**自觉爱国守法。忠于祖国，忠于人民，恪守[宪法](https://www.pkulaw.com/chl/7c7e81f43957c58bbdfb.html?way=textSlc)原则，遵守法律法规，依法依规开展托育服务。不得损害国家利益、社会公共利益、违背社会公序良俗。

　　**三、**传播优秀文化。传承中华传统美德和优秀文化，践行社会主义核心价值观，培养婴幼儿良好品行和习惯。不得传播有损婴幼儿健康成长的不良文化。

　　**四、**注重情感呵护。敏感观察，积极回应，尊重个体差异，关心爱护每一位婴幼儿，形成温暖稳定的关系。不得忽视、歧视、侮辱、虐待婴幼儿。

　　**五、**提供科学照护。遵循婴幼儿成长规律，合理安排每日生活和游戏活动，支持婴幼儿主动探索、操作体验、互动交流和表达表现。不得开展超出婴幼儿接受能力的活动。

　　**六、**保障安全健康。创设安全健康的环境，熟练掌握安全防范、膳食营养、疾病防控和应急处置等方面的知识和技能。不得在紧急情况下置婴幼儿安危于不顾，自行逃离。

　　**七、**践行家托共育。注重与婴幼儿家庭密切合作，保持经常性良好沟通，传播科学育儿理念，提供家庭照护指导服务。不得滥用生长发育测评等造成家长焦虑。

　　**八、**提升专业素养。热爱托育工作，增强职业荣誉感，加强业务学习，做好情绪管理，提高适应新时代托育服务发展要求的专业能力。不得有损害职业形象的行为。

　　**九、**加强团队协作。尊重同事，以诚相待，相互支持，充分沟通婴幼儿信息，协同开展照护活动，不断改进和提升服务质量。不得敷衍塞责、相互推诿、破坏团结。

　　**十、**坚守诚信自律。诚实守信，严于律己，尊重婴幼儿及其家庭的合法权益，自觉遵守托育服务标准和规范。不得收受婴幼儿家长礼品或利用家长资源谋取私利。

#### 本篇引用的法规

##### 中央法规

[中共中央、国务院关于优化生育政策促进人口长期均衡发展的决定](https://www.pkulaw.com/chl/1247bc81dccaeed3bdfb.html?way=textRightFblx)

[国务院办公厅关于促进3岁以下婴幼儿照护服务发展的指导意见](https://www.pkulaw.com/chl/434b24b8b5769383bdfb.html?way=textRightFblx)

[中华人民共和国宪法(2018修正)](https://www.pkulaw.com/chl/7c7e81f43957c58bbdfb.html?way=textRightFblx)

©北大法宝：（[www.pkulaw.com](https://www.pkulaw.com/)）专业提供法律信息、法学知识和法律软件领域各类解决方案。北大法宝为您提供丰富的参考资料，正式引用法规条文时请与标准文本核对。 欢迎查看所有[产品和服务](http://www.pkulaw.net/)。
[法宝快讯： 如何快速找到您需要的检索结果？ 法宝 V6 有何新特色？](http://www.pkulaw.com/helps/69.html)

扫描二维码阅读原文

原文链接：<https://www.pkulaw.com/chl/bc77e6a4e1650b3fbdfb.html>
